# Impact of Amplification Efficiency Approaches on Telomere Length Measurement *via* Quantitative-Polymerase Chain Reaction

**DOI:** 10.3389/fgene.2021.728603

**Published:** 2021-09-17

**Authors:** Waylon J. Hastings, Dan T. A. Eisenberg, Idan Shalev

**Affiliations:** ^1^Department of Biobehavioral Health, The Pennsylvania State University, University Park, PA, United States; ^2^Department of Anthropology, University of Washington, Seattle, WA, United States

**Keywords:** telomere length, qPCR, amplification efficiency, external validity, LinRegPCR

## Abstract

**Background:** Precise determination of amplification efficiency is critical for reliable conversion of within-sample changes in fluorescence occurring on a logarithmic scale to between-sample differences in DNA content occurring on a linear scale. This endeavor is especially challenging for the telomere length (TL) quantitative-PCR (qPCR) assay, where amplification efficiency can vary between reactions targeting telomeric repeats (T) and those targeting a single-copy gene (S) to calculate TL as the T/S ratio.

**Methods:** We compared seven different approaches toward estimating amplification efficiency, including the standard-curve method utilized by the qPCR instrument software, and alternative approaches which estimate efficiency on a reaction-by-reaction basis using the stand-alone program LinRegPCR. After calculating T/S ratios using efficiency estimates from each approach (*N* = 363), we tested their relative performance on metrics of assay precision and correlates of external validity including chronological age (age range = 1–72 years), across tissues within-person (leukocyte-buccal), and between parents and offspring.

**Results:** Estimated amplification efficiency for telomere reactions was significantly lower than estimates for single-copy gene reactions. Efficiency estimates for both reaction sets were significantly higher when estimated with the standard-curve method utilized by the qPCR instrument relative to estimates reconstructed during the log-linear phase with LinRegPCR. While estimates of single-copy gene efficiency reconstructed using LinRegPCR measured within 90% of perfect exponential doubling (*E* = 1.92), estimates generated using the standard-curve method were inflated beyond 100% (*E* = 2.10–2.12), indicating poor fidelity. Despite differences in raw value, TL measurements calculated with LinRegPCR efficiency estimates exhibited similar relationships with external validity correlates to measurements generated using the qPCR instrument software.

**Conclusion:** Since methods to estimate amplification efficiency can vary across qPCR instruments, we suggest that future analyses empirically consider external methods of efficiency calculations such as LinRegPCR, and that already generated data be re-analyzed to glean possible improvements.

## Introduction

Telomeres are repetitive nucleoprotein regions at chromosome ends which prevent end to end fusions and protect remaining DNA from degradation. Large population studies have associated shorter telomere length (TL) with a range of health problems and shorter life expectancy (Wang et al., 2018), leading to their regard as a hallmark of biological aging (Lopez-Otin et al., 2013). Even so, technical challenges with TL measurement have led to questions regarding their utility as a biomarker of aging (Hastings et al., 2017).

The most common approach to quantify TL in epidemiological studies is quantitative-PCR (qPCR), which expresses telomeric repeats (T) relative to a single-copy gene (S) *via* the T/S ratio (Cawthon, 2002). A challenge in qPCR measurement is the conversion of within-sample changes in fluorescence, which occur on a logarithmic scale, to between-sample differences in DNA content occurring on a linear scale. Specifically, qPCR methods estimate differences in the amount of target nucleotide (e.g., T or S) between experimental samples based on the number of cycles each sample takes to reach a fluorescence threshold, also called the quantification cycle (Cq). Between-sample differences in target nucleotide, which tend to be relatively small in magnitude at baseline, increase exponentially as the target nucleotide is amplified throughout the qPCR assay, enabling easier differentiation.

The rate at which between-sample differences increase is dependent upon the efficiency of exponential amplification across qPCR cycles, which is typically estimated by monitoring changes in fluorescence using a DNA-binding dye such as SYBR Green I. In theory, the amount of target DNA or RNA template should double during each polymerase chain reaction (PCR) cycle. Under this assumption, a target molecule present in 100 copies at the end of a given qPCR cycle would be expected to host 200 copies by the end of the subsequent cycle. Similarly, a second sample with 75 copies of the target would have 150 copies by the end of the subsequent cycle, and the between-sample difference would double from 25 copies to 50 copies. An assumption of perfect amplification efficiency is reflected by the use of 2.0 as the base value within exponential functions used to calculate normalized gene expression *via* the ΔΔ-Ct method (Livak and Schmittgen, 2001), as well as sometimes TL *via* the T/S ratio (Lu et al., 2011; Prescott et al., 2011). However, perfect doubling across all qPCR cycles is unlikely. Many factors influence qPCR efficiency including sequence and concentration of primers, carryover chemicals from sample or DNA processing, and temperature settings on the qPCR apparatus (Bustin et al., 2009). Therefore, accurately estimating amplification efficiency is critical for precise determination of between-sample differences using qPCR.

The most commonly implemented approach to estimate amplification efficiency is to construct a standard-curve using a serial dilution of samples with known amounts of target nucleotide template (Bustin et al., 2009). Once the qPCR assay has completed, a plot is generated that compares the Cq values for each standard vs. the logarithm of their known baseline concentration. Since fluorescence intensity is proportional to the amount of target nucleotide, the amount of target sequence present upon reaching the fluorescence threshold is equivalent across all standards. Thus, differences in the number of cycles it takes for each standard to reach this threshold, i.e., differences in Cq values, are a direct function of differences in baseline concentrations and the amplification efficiency. This relation is expressed mathematically as $E=10^{-}\,\left(\frac{1}{M}\right)$, where E is the amplification efficiency and *M* is the slope of the line comparing Cq values to known log_10_-concentrations of standards^1^.

Although widely utilized, efficiency estimates generated using a standard-curve can vary by up to 40% depending on the number of standards utilized, standard concentration, the number of technical replicates, errors in dilutions, and the specific qPCR instrument used (Svec et al., 2015). Variability due to differences in instrumentation is particularly salient, since rules for how background fluorescence is mitigated, and hardware related to excitation and emission (e.g., filters, slit width, and exposure time), vary between instruments. Even so, most researchers still default to instrumentation software when conducting qPCR analysis (Rebrikov and Trofimov, 2006), a potentially problematic norm for the telomere field given how small differences in efficiency estimates may be exponentially propagated into errors in baseline concentration estimation (Ruijter et al., 2009).

Due to these and other concerns, alternative standard-free means of efficiency estimation have been developed. One alternative approach is to evaluate patterns of fluorescence on a reaction-by-reaction basis, as in the stand-alone program LinRegPCR (Ramakers et al., 2003). In this method, the fluorescence pattern of each reaction is inspected during the log-linear phase, the duration of the qPCR assay after early cycles when fluorescence is below or near detection limits and before later cycles when limiting reagents are exhausted and fluorescence plateaus. During this phase, the efficiency (*E*) can be estimated from the slope of a line relating cycle number to log_10_-fluorescence as *E* = 10^*Slope*^. Using a common range of fluorescence values referred to as the window-of-linearity^2^, this process is repeated to determine the individual amplification efficiencies for all reactions on a qPCR assay. Averaging across technical replicates gives the efficiency per sample, and averaging across all reactions gives the efficiency at the plate-level, as with the standard-curve method.

Concerns expressed about accurate measurement of amplification efficiency for qPCR more broadly (Bustin et al., 2009), have yet to receive substantial attention in telomere research. In fact, details related to PCR efficiency are the least commonly reported metric in comparative studies utilizing TL measurements generated using qPCR (Lindrose et al., 2021). This oversight is concerning, given the increased susceptibility of TL measurements generated *via* qPCR to variability in amplification efficiency. The T/S ratio is calculated using the formula T/S=(ETC⁢qTESC⁢qS)-1, where *E*_*T/S*_ is the efficiency of exponential amplification for reactions targeting telomeric repeats or the single-copy gene, respectively, and Cq_*T/S*_ is the cycle at which a given replicate targeting telomeric repeats or the single-copy gene reaches the critical threshold of fluorescence quantification. Therefore, precise determination of between-sample differences in T/S ratio measurements requires accurate assessment of telomeric and single-copy gene reaction efficiencies, a challenging endeavor that can contribute to concerns about the precision of TL measurement *via* qPCR relative to other techniques such as Southern Blot (Aubert et al., 2012; Nettle et al., 2021).

To explore the impact of qPCR amplification efficiency on TL measurements, we compared observed qPCR efficiency values generated using the standard-curve approach with software built into the Rotor-Gene Q real-time qPCR instrument with efficiency estimates determined using the external analysis program LinRegPCR (Ramakers et al., 2003). Further, we tested how accounting for such differences impacts the precision and external validity of resulting TL measurements.

## Method

### DNA Extraction and Telomere Length Assessment

Whole blood and/or buccal epithelial cells were collected from 261 individuals spanning four generations: 25 grandmothers (age 52.6–72.2), 108 mothers (age 29.1–43.6) and 127 children (46.4% male; age 0.5–24.9), and 1 great grandchild (female; age = 3.6) as part of an ancillary project within the Female Growth and Development Study (FGDS) investigating intergenerational transmission of trauma *via* differences in TL (Etzel et al., 2020; Hastings et al., 2020). Blood draws were not mandatory for participation in FGDS, nor were they collected from participants aged 18 or younger, and a large proportion of participants elected to provide samples only *via* buccal swabs. DNA for TL analyses was extracted from buffy coat (*N* = 94; 12 grandmothers, 79 mothers, and 3 children) and buccal epithelial cells (*N* = 270; 26 grandmothers, 116 mothers, 127 children, and 1 great grandchild; dataset included multiple time points for 1 grandmother and 8 mothers) using QIAamp DNA Mini Kits (Qiagen, Germany). DNA purity and quality was assessed using 260/230 and 260/280 ratios, but no exclusionary criteria were imposed prior to assays. DNA was stored at −80°C until TL analysis. All TL assays were performed by WJH on a Qiagen Rotor-Gene Q thermocycler, using a standard qPCR protocol (Cawthon, 2002). Each telomere assay comprised two qPCR runs, one run quantifying telomere repeats (T) and a second run quantifying genome copy number (S) using the single-copy gene *36B4.* Detailed descriptions of sample handling and processing, as well as details regarding qPCR assay and quality control are summarized in the Supplementary Table 1 in accordance with guidelines recommended by the Telomere Research Network (doi: 10.31219/osf.io/9pzst). The same DNA aliquot was used for T and S runs. Each run hosted triplicate reactions of 22 samples, 5 standard-curve samples, and 6 positive controls on 100-well disks. Standard-curves consisted of a series of five ten-fold dilutions of double-stranded oligomers mimicking telomeric or single-copy gene sequences. Oligomers for the telomere standard-curve were 84 bp long and comprised 16 repeats of the canonical telomere sequence in humans (TTAGGG). Oligomers for the single-copy gene standard-curve consisted of a double-stranded oligomers comprising a 75 bp tract of the *36B4* gene. Sequences for oligomer standards are provided in Supplementary Table 1.

### Amplification Efficiency Estimation and T/S Ratio Calculation

Telomere length was quantified as the T/S ratio, calculated as T/S=(ETC⁢qTESC⁢qS)-1, where *E*_*T/S*_ is the efficiency of exponential amplification for reactions targeting telomeric repeats or the single-copy gene, respectively, and Cq_*T/S*_ is the cycle at which a given replicate targeting telomeric repeats or the single-copy gene reaches the critical threshold of fluorescence quantification. Seven different sets of T/S ratios were generated, each utilizing a different approach toward estimating amplification efficiency (Table 1). These measurements included T/S ratios calculated using efficiency estimates using the standard-curve generated by the Rotor-Gene Q instrument software (Version 2.1.0) for each plate independently (T/S_RotorGeneCurve_). Given work showing decreased assay variability when efficiencies are aggregated at the amplicon level across a given batch of plates (Čikoš et al., 2007), we also calculated efficiency estimates based on the average of standard-curve-based efficiencies across all T plates and across all S plates (T/S_RotorGeneBatch_). The remaining estimates were generated using LinRegPCR Version 2020.1 (Ramakers et al., 2003). LinRegPCR efficiency estimates differed on whether they were allowed to vary for each individual replicate (T/S_LinRegRep_), were aggregated across triplicates at the sample-level (T/S_*LinRegSamp*_), across samples at the plate-level (T/S_LinRegPlae_), or across plates at the amplicon-level (T/S_LinReg__Batch_). A final set of T/S ratio estimates were generated by reconstructing the standard-curve using known standard concentrations and Cq values from LinRegPCR (T/S_LinRegCurve_). This last implementation used the same method as T/S_RotorGeneCurve_, varying only in that the Cq values used were those produced by LinRegPCR instead of those determined by the Rotor-Gene Q instrument.

**TABLE 1:** Summary of T/S ratio calculation approaches.

**Notation**	**Elaboration**
T/S_RotorGeneCurve_	T/S ratios generated using Cq values from the Rotor-Gene Q instrument and plate-level efficiency estimates from the Rotor-Gene Q instrument. Plate-level efficiency estimates are automatically calculated by the Rotor-Gene Q instrument using the standard-curve for each plate.
T/S_RotorGeneBatch_	T/S ratios generated using Cq values from the Rotor-Gene Q instrument and amplicon-level efficiency estimates from the Rotor-Gene Q instrument. Amplicon-level efficiency estimates for telomere reactions was calculated as the average of efficiency estimates generated using standard-curves across all T plates. Similarly, amplicon-level efficiency estimates for single-copy gene reactions was calculated as the average of efficiency estimates generated using standard-curves across all S plates.
T/S_LinRegRep_	T/S ratios generated using Cq values and replicate-level efficiency estimates from LinRegPCR.
T/S_LinRegSamp_	T/S ratios generated using Cq values and sample-level efficiency estimates from LinRegPCR. Sample-level efficiency calculated as the average efficiency across technical replicates within a plate.
T/S_LinRegPlate_	T/S ratios generated using Cq values and plate-level efficiency estimates from LinRegPCR. Plate-level efficiency calculated as the average efficiency across all analytical samples and controls within a plate (i.e., not including standards or H_2_O blank).
T/S_LinReg__Batch_	T/S ratios generated using Cq values and amplicon-level efficiency estimates from LinRegPCR. Amplicon-level efficiency is automatically estimated by LinRegPCR as the average efficiency for a given amplicon (T or S) across all reactions on all plates.
T/S_LinRegCurve_	T/S ratios generated using Cq values and plate-level efficiency estimates from LinRegPCR. In this instance, plate-level efficiency was calculated by reconstructing a standard-curve using known standard concentrations and Cq values estimated by LinRegPCR. Efficiency was calculated using the equation $E=10^{-}\,\left(\frac{1}{M}\right)$, where E is the amplification efficiency and M is the slope of the line comparing Cq values (*y*-axis) to known log_10_-concentrations of standards (*x*-axis)

*The same fluorescence threshold was used across all T plates and all S plates to determine quantification cycle (Cq) values from the Rotor-Gene Q instrument (0.4996). Raw fluorescence from all plates was extracted and compiled for analysis in LinRegPCR as a single batch. In this pipeline, standards were clustered into amplicon groups distinct from samples and controls. The amplicon groups were G1, T standards; G2, T reactions and controls; G3, S standards; and G4, S reactions and controls. LinRegPCR establishes a different window of linearity for each amplicon group to calculate efficiency values, but uses the same threshold of detection for all groups when generating Cq values (0.4260).*

The same quantification threshold was used across all plates to determine Cq values from the Rotor-Gene Q instrument (0.4996). Raw fluorescence values from all plates were compiled and processed as one batch using LinRegPCR. In this pipeline, distinct amplicon groups were specified for single-copy gene reactions and telomere reactions, as well as for standards relative to analytical samples and controls. The specific amplicon groups were G1, telomere oligomer standards; G2, telomere analytical samples and positive controls; G3, single-copy gene oligomer standards; and G4, single-copy gene analytical samples and positive controls. During processing LinRegPCR allows the window-of-linearity to vary for each amplicon group, but uses the same quantification threshold for all groups when generating Cq values (0.4260).

### Sample Overview and Statistical Analyses

The present work summarizes data generated from TL assessments of 2,152 replicate reactions across 34 qPCR runs (17 T and 17 S). These replicates represent T and S reactions from 363 samples run in triplicate following removal of outlier replicates as described in Supplementary Table 1. One sample that failed to pass quality control criteria after two attempts was removed from all analyses. A full description of sample flow and subsets used in each analysis is provided in Supplementary Figure 1. Differences in plate-level efficiency estimates, coefficient of variation (CV) across replicate T-estimates and S-estimates, and sample-level T/S ratio values were assessed using paired sample *t*-tests. T-estimates and S-estimates were natural-log transformed prior to calculating CV across technical replicates to better approximate normality. T/S ratios were calculated using raw T-estimates and S-estimates (i.e., not natural-log transformed). Tests for differences in sample CV were conducted following the method of Feltz and Miller (1996) using the R package cvequality. Control for multiple comparisons was conducted using Bonferroni adjusted *p*-values where appropriate.

To better understand how approaches toward amplification efficiency calculation would influence the findings derived from TL data, we compared how T/S ratios constructed using the different approaches varied in relation to external validity correlates, including chronological age, across-tissues within person, and among parents and offspring (Eisenberg, 2016). Differences in T/S ratio correlation coefficients were evaluated based on overlap of 83.4% confidence intervals (CIs; Knol et al., 2011). Statistical analyses were conducted with IBM SPSS Statistics 26. Sample size estimates for reported power calculations were performed in R 4.0.2 using the “pwr.r.test” command with power = 0.80, α = 0.05, and effect size equal to the observed correlation coefficient. Simulation analyses were conducted in Stata 14.1.

## Results

### Amplification Efficiency Estimates

The estimated amplification efficiency for reactions targeting telomeric repeats was significantly lower than the efficiency of reactions targeting the single-copy gene, irrespective of whether these estimates were generated using the Rotor-Gene Q standard-curve, LinRegPCR standard-curve, or reconstructed during the log-linear phase with LinRegPCR (Table 2). For T reactions and S reactions, estimates of reaction efficiency were significantly higher when estimated using the Rotor-Gene Q standard-curve relative to either LinRegPCR approach (Table 2).

**TABLE 2:** Differences in plate-level amplification efficiency as a function of amplicon target and calculation approach.

**Approach**	**T plate efficiency**	**S plate efficiency**	***p*-value (between amplicons)**
(1) Rotor-gene Q standard-curve	1.89 (0.03)	2.12 (0.04)	1.40E−12
(2) LinRegPCR standard-curve	1.83 (0.04)	2.10 (0.05)	4.55E−11
(3) LinRegPCR reconstructed during exponential phase	1.86 (0.05)	1.92 (0.05)	2.57E−08
*p*-value between approaches (1 vs. 2)	7.51E−08	2.98E−04	
*p*-value between approaches (2 vs. 3)	0.129	1.02E−08	
*p*-value between approaches (1 vs. 3)	0.048	1.34E−09	

*Efficiency values are mean (standard deviation) across 17 T plates or 17 S plates. Reported *p*-values are results of paired sample *t*-tests across 17 pairs of quantitative-PCR (qPCR) plates.*

### Precision Across Technical Replicates

To investigate the impact of efficiency estimation approaches on assay precision, we compared the CV across replicate natural-log transformed T-estimates (Ln[ETC⁢qT]) and natural-log transformed S-estimates (Ln[ESC⁢qS]) calculated using the various efficiency approaches. The CV across replicate T-estimates was significantly larger for values generated using replicate-level efficiencies relative to all other approaches, indicating greater variability and/or measurement error when using this method (Supplementary Tables 2, 3). Similar findings were observed for the CV across replicate S-estimates (Supplementary Tables 4, 5). Furthermore, the CV across replicate S-estimates was significantly larger when generated using a batch-level efficiency estimate (i.e., RotorGeneBatch and LinRegBatch) relative to estimates using the standard-curve for each plate, irrespective of whether the standard-curve was calculated using the Rotor-Gene Q instrument or LinRegPCR. Full results of analyses of CV across replicate natural-log transformed T and S estimates are presented in Supplementary Tables 2–5.

### T/S Ratio Estimates

Using different approaches toward amplification efficiency estimation significantly impacted the scale of T/S ratio values for the entire sample (Table 3). T/S ratio values were significantly different in nearly all pairwise contrasts, excepting T/S ratios calculated using replicate-level and sample-level efficiencies, which did not differ significantly after controlling for multiple comparisons (Supplementary Table 6). Similar findings were observed in independent analyses of buccal samples. However, T/S ratios for leukocyte samples tended to be more closely related across the LinRegPCR-based approaches. Full test statistics and *p*-values for pairwise comparisons of T/S ratios are reported in Supplementary Table 6.

**TABLE 3:** T/S ratios calculated using different amplification efficiencies.

	T/S_RotorGeneCurve_	T/S_RotorGeneBatch_	T/S_LinRegRep_	T/S_*LinRegSamp*_	T/S_LinRegPlate_	T/S_*LinRegBatch*_	T/S_*LinRegCurve*_
All samples	5.39 (2.49)	5.23 (2.41)	0.99 (1.10)	0.94 (0.94)	0.73 (0.31)	0.74 (0.30)	9.71 (4.22)
Leukocyte only	5.71 (2.20)	5.56 (2.14)	1.24 (1.47)	1.17 (1.20)	0.79 (0.29)	0.91 (0.26)	10.31 (3.90)
Buccal only	5.28 (2.58)	5.11 (2.49)	0.90 (0.92)	0.87 (0.81)	0.71 (0.32)	0.72 (0.31)	9.50 (4.32)

*Mean and standard deviation of T/S ratio values calculated using different amplification efficiencies for the whole sample and for leukocyte samples and buccal samples independently.*

To better understand the impact of different efficiency estimation approaches on the distribution of T/S ratios, we calculated the CV of T/S ratios within the analytical sample for each approach (Supplementary Table 7). The largest dispersion of values was observed for T/S ratios calculated using replicate-level and sample-level efficiencies, which had CV greater than 100% within the sample. By contrast, the CV of T/S ratios calculated with efficiency estimates aggregated at the plate and amplicon levels tended to be similar regardless of the approach, varying between 40 and 46%. Full test statistics and *p*-values for pairwise comparisons of T/S ratio CV are reported in Supplementary Table 8.

### External Validity Correlates

After testing for differences in scale and distribution, we explored interrelations among T/S ratios calculated using different efficiency estimates, and evaluated their relationship to external validity correlates. Despite differences in mean value, most T/S ratios generated using the different efficiency estimation approaches were very highly correlated with one another (*r* > 0.90; Supplementary Table 9). Exceptions to this were T/S ratios generated using LinRegPCR replicate-level and sample-level efficiency estimates. T/S ratios calculated using these two approaches were highly correlated with one another (*r* = 0.957), but were weakly correlated with T/S ratios generated using all other approaches (*r* = 0.100–0.376).

**FIGURE 1:**
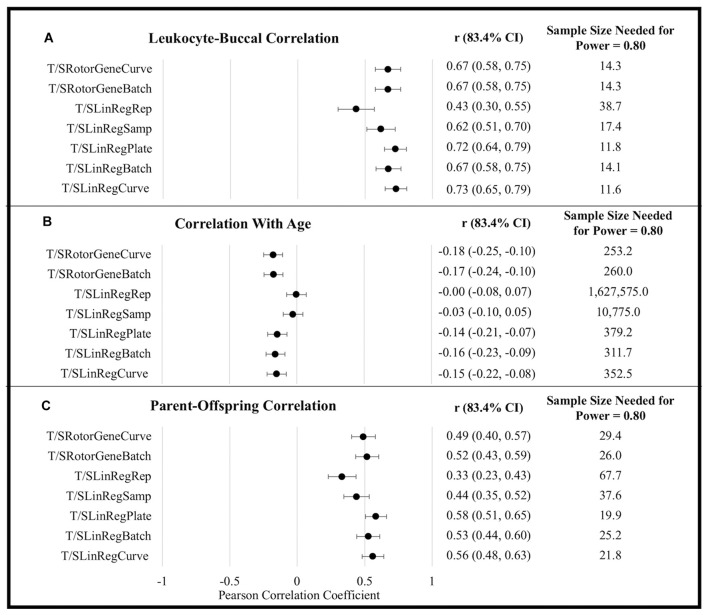
External validity metrics for T/S ratios calculated using different amplification efficiencies. **(A)** Within-person Pearson correlation between leukocyte and buccal sample T/S ratios collected at the same time point. Correlations calculated controlling for sex and age. **(B)** Pearson correlations between age and T/S ratios. Correlations calculated controlling for sex and tissue (leukocyte/buccal). **(C)** Pearson correlation between T/S ratios of parent and offspring pairs. Correlations calculated controlling for offspring sex, parental age, offspring age, and tissue (leukocyte/buccal). CI, confidence interval.

T/S ratios generated using efficiency estimates aggregated at the level of a single plate (i.e., T/S_RotorGeneCurve_, T/S_LinRegPlate_, and T/S_LinRegCurve_) tended to exhibit the strongest correlations with all metrics of external validity (Figure 1). Although differences in correlation coefficients were not statistically significant, T/S ratios generated using LinRegPCR tended to perform better with respect to correlations between tissues and among parents and offspring. By contrast, T/S ratios generated using the Rotor-Gene Q standard-curve tended to exhibit larger correlations with chronological age. Similar differences in the association with chronological age were observed when leukocyte and buccal samples were analyzed independently (Supplementary Table 10).

#### Simulation Analysis

To further evaluate the different approaches, we conducted a simulation analysis with external validity correlates. Based on prior literature, we expected the cross tissue results to be most informative, followed by parent-offspring, with chronological age to be least informative. To test this hypothesis, we created a simulated TL variable with a sample size of 200 that correlated with a simulated “age” variable at *r* = 0.15 and a simulated “tissue” variable at *r* = 0.70. We then created a second “noisy” TL variable that correlated with the original TL variable at *r*≈0.87. Finally, we compared how well the noisy and non-noisy TL variables correlated with age and tissue to determine whether the magnitude of improvement was greater for the correlation with age or the correlation with tissue. This comparison is expressed by the formula f(x)=(rN⁢o⁢n-n⁢o⁢i⁢s⁢y⁢T⁢L↔T⁢I⁢s⁢s⁢u⁢erNoisyTL↔Tissue)(rN⁢o⁢n-n⁢o⁢i⁢s⁢y⁢T⁢L↔A⁢g⁢erN⁢o⁢i⁢s⁢y⁢T⁢L↔)A⁢g⁢e. If this expression is positive, then the magnitude of improvement was greater for the correlation between TL and tissue than for the correlation between TL and age. This was observed in 930 out of 1,000 simulations with *mean*_*f*(*x*)_ = 0.079 ± 0.04, confirming expectations (Supplementary Figure 2). Stata Code for the simulation analysis is provided in the Supplementary Material.

#### Power Analysis

To explore power yielded from using the various approaches, we compared the sample sizes needed to distinguish T/S ratio external validity correlates as significantly different from zero (α = 0.05, power = 0.80). For example, to detect the correlation of TL across tissues using Rotor-Gene Q estimates (r_Average_ = 0.670) requires an average sample size of 14.30, while an average sample size of 12.49 is required to detect an effect using LinRegPCR estimates implemented at the plate or batch level (r_Average_ = 0.709). This equates to being able to detect a significant effect with a 12.66% smaller sample size. By contrast, the sample size needed to detect a significant association with chronological age was 26.22% smaller using Rotor-Gene Q efficiency estimates (r_Average_ = −0.174) relative to T/S ratios generated using LinRegPCR (r_Average_ = −0.150). Following the same procedure for parent-offspring correlations yields an estimated 19.63% reduction in sample size for T/S ratios generated using LinRegPCR relative to T/S ratios generated using the Rotor-Gene Q instrument.

## Discussion

Telomere length assessment *via* qPCR is subject to bias from a host of analytical and pre-analytical factors [reviewed in Lin et al. (2019)], leading some to challenge the utility of telomeres as a biomarker of aging (Boonekamp et al., 2013). Nevertheless, TL measurement *via* qPCR remains widely used in telomere research. Thus, elucidating measurement practices which enhance reproducibility and precision is of great interest.

In the current work we compared seven different approaches toward estimating amplification efficiency (Table 1), and explored their relative impact on metrics of assay precision and external validity. Our results show significantly lower amplification efficiency in reactions targeting telomeric repeats relative to those targeting the single-copy gene *36B4* (Table 2). These differences could result from the highly repetitive nature of the telomeric region, or the imperfect match between this region and the telomere primer set (the imperfect match is intentional clever primer design to allow quantification of this repetitive region). Even so, estimated efficiency of telomeric reactions was greater than 90% (>1.80), a recommended benchmark for validating qPCR (Svec et al., 2015). Estimated efficiency of telomeric and single-copy gene reactions were both significantly higher when calculated using the standard-curve approach with the Rotor-Gene Q software relative to estimates generated using LinRegPCR, leading to significant differences in resulting T/S ratio values (Table 3 and Supplementary Table 6).

Differences in the magnitude of T/S ratio values serve to illustrate how even small variation in amplification efficiency can substantially impact the scale of TL measurements, which limits cross-sectional studies and cross-lab comparisons. Efficiency estimates for T reactions generated by the Rotor-Gene Q standard-curve varied by less than 4% from those estimated by the standard-curve implemented using LinRegPCR, and efficiency estimates for S reactions varied by less than 1% between the two approaches. However, the difference in average T/S ratio value between these two methods was nearly 80%.

Despite differences in scale, most efficiency estimation approaches did not substantial impact the distribution of T/S ratio values within the analytical sample. For example, T/S_RotorGeneCurve_ and T/S_RotorGeneBatch_ estimates were over fivefold larger than T/S_LinRegPlate_ and T/S_*LinRegBatch*_ estimates, but all four exhibited similar CV within the analytical sample (Supplementary Tables 7, 8). TL measurements generated using the standard-curve were also highly correlated to T/S ratios calculated using log-linear estimates from LinRegPCR, so long as log-linear estimates were aggregated at the plate or amplicon level (Supplementary Table 9). By contrast, T/S ratios generated with replicate-level and sample-level efficiencies were weakly correlated to T/S ratios generated using other approaches, had significantly larger CV across replicate T estimates and S estimates, and tended to exhibit significantly weaker correlations in analyses of external validity correlates (Figure 1). Taken together, these observations serve to illustrate how LinRegPCR efficiency estimates implemented at replicate and sample levels can lead to diminished performance, as has been previously reported (Ruijter et al., 2009).

Our results demonstrate substantial overlap among qPCR assay measures of external validity using estimates of amplification efficiency implemented at the plate-level (i.e., T/S_RotorGeneCurve_, T/S_LinRegPlate_, and T/S_LinRegCurve_) and amplicon-level (i.e., T/S_RotorGeneBatch_ and T/S_LinReg__Batch_). Notably, this observation holds for estimates reconstructed using changes in the log-linear phase with LinRegPCR instead of relying on patterns of fluorescence change across serially diluted standards, as is commonly done for built-in instrumentation software. Serially diluted standards could underperform in estimation of efficiency values because of greater statistical noise, since standards are systematically different sample types than measured samples, due to dilution errors, or because of PCR inhibitors or enhancers which cause amplification performance to vary with dilutions. In this instance, efficiency estimates for the single-copy gene generated using the standard-curve with either LinRegPCR or the Rotor-Gene Q were measured as being higher than the hypothetical ideal value of 2.0, which can indicate the presence of contaminants inhibiting the activity of the DNA polymerase for higher concentrated standards. By contrast, efficiency estimates reconstructed on a reaction-by-reaction basis for the single-copy gene were only 1.92 on average, much closer to values observed for reactions targeting telomeric repeats. This observation, combined with observed similarities in precision and slightly higher performance of LinRegPCR across metrics of external validity, lead us to suggest that data be analyzed using an external program such as LinRegPCR instead of relying on instrumentation software. Doing so may help decrease variation across labs (Martin-Ruiz et al., 2015) which could partially result from differences in analysis settings across instruments (Svec et al., 2015). However, we caution that these results were only obtained after careful validation of our assay by monitoring standard-curve-based efficiency and R^2^ estimates. Until LinRegPCR is better established, we recommend that other laboratories use standard-curve measures as a method of assay validation before attempting LinRegPCR standard-curve-free methods. Furthermore, we note that results obtained here, although consistent with previous reports, may not generalize to studies in other populations or using different qPCR-based methodology for TL measurement.

Most epidemiological studies utilizing TL measurements generated using qPCR either rely on instrument software to estimate amplification efficiency based on a standard-curve, or do not report how efficiency estimates are derived. According to one study, PCR efficiency is the least commonly reported metric in comparative studies utilizing TL measurements generated using qPCR (Lindrose et al., 2021). These norms of practice run counter to new guidelines issued by the Telomere Research Network for studies using qPCR (doi: 10.31219/osf.io/9pzst), as well as the MIQE guidelines for real-time qPCR experiments more broadly (Bustin et al., 2009). Thus, increased rigor in the reporting of data analysis and processing is needed, and may help mitigate problems with precision and reproducibility in epidemiological studies using qPCR-based estimates of TL.

We note that empirically evaluating T/S measures calculated with different methods of efficiency calculations can be applied not just to future work, but also to existing datasets (as long as raw fluorescence values from qPCR run files remain available). We suggest that such re-analysis calibrate with measures of external validity as reported here. Optimizing based on measures of external validity is likely to result in improved measurement accuracy—however, such optimization does mean that these correlations with measures of external validity need to be interpreted with caution since they are likely to be somewhat overfit. While the suggested LinRegPCR analysis method does require some extra steps, this labor may be worth it for the increased consistency across studies when using an external method instead of relying on instrumentation software that is likely to vary between labs. It is easier, cheaper and more respectful of subjects to make the most of the data we have rather than to have to collect larger sample sizes to accomplish our research goals.

## Data Availability Statement

The original contributions presented in the study are publicly available. These data can be found in the data repository site figshare at https://doi.org/10.6084/m9.figshare.13872683.v2. Further inquiries can be directed to the corresponding authors.

## Ethics Statement

The studies involving human participants were reviewed and approved by the Institutional Review Board at The Pennsylvania State University. Written informed consent to participate in this study was provided by the participants’ legal guardian/next of kin.

## Author Contributions

WH conceived the original study, conducted the telomere length assays, and performed the statistical analyses. IS secured the funding for telomere length assays. WH and DE conceived the analytical approach. WH, IS, and DE wrote the article. All authors approved the submitted version.

## Conflict of Interest

The authors declare that the research was conducted in the absence of any commercial or financial relationships that could be construed as a potential conflict of interest.

## Publisher’s Note

All claims expressed in this article are solely those of the authors and do not necessarily represent those of their affiliated organizations, or those of the publisher, the editors and the reviewers. Any product that may be evaluated in this article, or claim that may be made by its manufacturer, is not guaranteed or endorsed by the publisher.

## Footnotes

1 For a full derivation of this formula we refer readers to the excellent explanation by Svec et al. (2015) *Biomolecular Detection and Quantification, 3*, 9–16.

2 For a full review of the methodology implemented in the LinRegPCR program see the original publication by the program developers Ramakers et al. (2003) *Neuroscience Letters, 339*(1), 62–66.
