# Supplementary material for: Impact of Amplification Efficiency Approaches on Telomere Length Measurement via Quantitative-Polymerase Chain Reaction
Source: Front Genet. 2021 Sep 17;12:728603. doi: 10.3389/fgene.2021.728603 (PMC8484782; doi:10.3389/fgene.2021.728603)
Supplement: Supplementary file 1 [file Data_Sheet_1.docx]

**Supplementary Table S1: Telomere Research Network Reporting Guidelines**

| **ITEM** | **DESCRIPTION** |
| --- | --- |
| **Sample Type, Storage, Extraction, and Integrity** | |
| Sample type | DNA samples were extracted from buffy coat isolated from whole blood collected via 10mL EDTA blood tubes (n=94; 12 grandmothers, 79 mothers, & 3 children) and buccal epithelial cells collected using buccal swabs (Isohelix) (270; 26 grandmothers, 116 mothers, & 127 children & 1 great-grandchild) |
| Sample storage conditions, including temperature, duration, and buffer | Buffy coat cells were stored at -80°C in a solution consisting of phosphate buffered saline (PBS) pH 7.2+EDTA (2mMol) + bovine serum albumin (BSA; 0.5%) prior to extraction. Buccal swabs were stored dry (no buffer) in snap top tubes at -80 prior to extraction. Duration between sample collection and DNA extraction ranged from 3.3 to 0.2 years with an average of 1.8 years. |
| DNA extraction method | QIAmp DNA Minikit (Qiagen) with no modification from factory guidelines. |
| DNA storage conditions, including freeze-thaw cycles | DNA was stored -80°C in QIAmp DNA Minikit ‘Buffer AE’. On average there were two freeze thaws for DNA samples between extraction and the qPCR assay. The first was to determine DNA concentration using Quant-iT PicoGreen reagent (Qiagen) and the second was to perform a dilution for the qPCR assay. Samples needed to be reassessed on qPCR assays (n=9) were thawed one additional time. Samples were stored for an average of 7.7 months between extraction and the PicoGreen assay and for another 10.5 months between the PicoGreen assay and qPCR assay. |
| Method of documenting DNA quality and integrity | DNA was quantified using Quant-iT PicoGreen Reagent (mean_buccal_=7.83 ng/uL; mean_leukocyte_=16.26ng/uL). DNA purity and quality was assessed using 260/230 and 260/280 ratios for all buccal samples and most leukocyte samples (N= 73; 78%), but no exclusionary criteria was imposed prior to assays. The averages for each tissue are provided below.  260/230_Leukocyte_ = 2.54; 260/280_Leukcoyte_ = 1.82  260/230_Buccal =_ 2.31; 260/280_Buccal_ = 1.84  DNA integrity was also assessed on additional subset of buccal (n=21; 7.8%) and leukocyte (n=7; 7.4%) prior to article submission using an Agilent 2200 Bioanalyzer. An average of 3.97 years had passed between DNA extraction and DNA integrity analyses. The average DNA Integrity Number (DIN) for buccal samples was 5.04 indicating moderate degradation. The average DIN for leukocyte samples was 9.17 indicating little to no degradation. |
| Percentage of samples specifically tested for DNA quality and integrity | 260/280 and 260/230 ratios were assessed on 94.2% of samples. Only 7.7% of samples were assessed using the Agilent 2200 Bioanalyzer. |
| **qPCR Assay** | |
| Method (qPCR, MMqPCR, aTL, etc.) | qPCR, wherein each telomere assay comprised two qPCR runs, one run quantifying telomere content (T) and a second run quantifying genome copy number (S) using the single copy gene *36B4.* The two runs (T & S) were always performed on the same day using the same DNA aliquot which was stored at room temperature between runs (~2.5 hours). Each run hosted triplicate reactions of 22 samples, 5 standards, and 6 positive controls on 100 well disks. qPCR assays were conducted across a period of 2.27 months. |
| PCR machine type | Qiagen Rotor-Gene Q using 100 well disks |
| Source of master mix and reagents, and final reaction volume | The final reaction mix for the telomeric DNA contains 1x QuantiTect SYBR Green Master Mix (Qiagen), 0.2U Uracil Glycosylase (Thermo Fisher Scientific), 0.1 u M forward primer, 0.1 u M reverse primer, and 3 ng DNA in a 20 u L reaction.  The reaction mix for 36B4 contains 1x QuantiTect SYBR Green Master Mix, 0.2U Uracil Glycosylase, 0.3 u M forward primer, 0.5 u M reverse primer, and 3 ng DNA in a 20 u L reaction. |
| Telomere primer sequences and concentration | Primers are purchased from IDT ([www.idtdna.com](http://www.idtdna.com)) in lab-ready format (HPLC purified, 100uM in IDTE Buffer pH 8.0).  Forward Primer: 5'CGGTTTGTTTGGGTTTGGGTTTGGGTTTGGGTTTGGGTT3′  Reverse Primer: 5'GGCTTGCCTTACCCTTACCCTTACCCTTACCCTTACCCT3′ |
| Single copy gene name, primer sequences, and concentration | Single copy gene was *36B4* gene encoding a ribosomal phosphoprotein. Primers are purchased from IDT ([www.idtdna.com](http://www.idtdna.com)) in lab-ready format (HPLC purified, 100uM in IDTE Buffer pH 8.0).  *36B4* Forward Primer 5'CAGCAAGTGGGAAGGTGTAATCC3′  *36B4* Reverse Primer 5'CCCATTCTATCATCAACGGGTACAA3′ |
| Full PCR program description including temperature, times, and cycle numbers | 50 °C – 2min  95 °C – 15min  95 °C – 15s followed by 60 °C for 1 min (data acquisition); 45 cycles. |
| PCR efficiency of single copy gene and telomere primers | LinRegPCR Amplicon-Level Efficiency Estimates  Telomere: 1.86  Single Copy Gene: 1.92  Rotor-Gene Q Batch-Level Efficiency Estimates  Telomere: 1.89  Single Copy Gene: 2.12 |
| Source of control samples | 6 positive controls were selected from within the sample such that each plate included control DNA extracted from blood of grandmothers, buccal of grandmothers, blood of mothers, buccal of mothers, and buccal of children. |
| Source and concentration of standard curve | Standard curves consisted of a series of five ten-fold dilutions of double stranded oligomers mimicking telomeric or single copy gene sequences. Oligomers are purchased from IDT ([www.idtdna.com](http://www.idtdna.com)) as lyophilized pellet with PAGE purification.  Double stranded oligomers for the telomere standard curve were 84bp long and comprised 16 repeats of the canonical telomere sequence in in humans (TTAGGG). A series of five ten-fold dilutions were performed such that each standard corresponded to 5.84x10^8^ – 5.84x10^4^ kb telomeric DNA per reaction.  Telomere Standard (Sense): 5’-CCC TAA CCC TAA CCC TAA CCC TAA CCC TAA CCC TAA CCC TAA CCC TAA CCC TAA CCC TAA CCC TAA CCC TAA CCC TAA CCC TAA -3’  Telomere Standard (Antisense): 5’- TTA GGG TTA GGG TTA GGG TTA GGG TTA GGG TTA GGG TTA GGG TTA GGG TTA GGG TTA GGG TTA GGG TTA GGG TTA GGG TTA GGG-3’  Double stranded oligomers for the single copy gene standard curve comprised a 75bp tract of the *36B4* gene. A series of five ten-fold dilutions were performed such that each standard corresponded to 1.33x10^6^ – 1.33x10^2^ genome copies per reaction.  Single Copy Gene Standard (Sense): 5’ -CAG CAA GTG GGA AGG TGT AAT CCG TCT CCA CAG ACA AGG CCA GGA CTC GTT TGT ACC CGT TGA TGA TAG AAT GGG -3’  Single Copy Gene Standard (Antisense) 5’- CCC ATT CTA TCA TCA ACG GGT ACA AAC GAG TCC TGG CCT TGT CTG TGG AGA CGG ATT ACA CCT TCC CAC TTG CTG -3’ |
| **Data Analysis** | |
| Mean and standard deviation or median range of telomere lengths | *Means below reflect calculations using LinRegPCR plate-level efficiencies. See Table 3 for T/S ratio mean by alternative approaches*  T/S ratio mean (SD) = 0.73 (0.31)  Buccal T/S ratio mean (SD): 0.71 (0.32)  Leukocyte T/S ratio mean (SD): 0.79 (0.29) |
| Number of sample replicates | Each sample was assessed for T and S on a single run with three replicates within the run. If the sample did not pass quality control criteria described below it was run a second time. |
| Level of independence of replicates | Replicates were drawn from the same DNA aliquot (i.e., the same tube). |
| Analytic method, considering replicate measurements, to determine final length | T and S estimates were calculated using the Cq_T/S_ values for individual replicates. T/S ratios were calculated using the average T and average S estimates across replicates. T/S ratios calculated using Rotor-Gene Q based efficiencies were computed using Cq values from the Rotor-Gene Q instrument. T/S ratios calculated using LinRegPCR based efficiencies were computed using Cq values determined by LinRegPCR. |
| Method of accounting for variation between replicates | When the standard deviation across replicate Cq values was greater than 0.25, replicate Cq values were evaluated based upon their deviation from mean Cq across triplicates. If one replicate deviated from the mean Cq by more than 15% it was considered an outlier and the mean Cq was recalculated using two replicates. This occurred for 15 T replicates and 11 S replicates among final sample. In the case where Cq standard deviation for either T or S replicates was still greater than 0.25 after removal of a single outlier, or was greater than 0.25 without a clear outlier defined by the criteria above, the sample was reassessed for both telomere content and genome copy number and subjected to the same quality control evaluation. A total of 9 samples were rerun a second time. |
| Method of accounting for well position effects within plates | The unique rotary design of the Rotor-Gene Q is optimized to minimize well position effects. As such no accounting for such effects was performed. |
| Method of accounting for between plate effects | 6 positive controls were selected from within the sample such that each plate included control DNA extracted from blood of grandmothers, buccal of grandmothers, blood of mothers, buccal of mothers, and buccal of children. For each plate, the T value and S value of each control DNA was divided by the average T value and S value for the same DNA across all runs to get a normalizing factor for that sample on a given plate. This was repeated for all controls on a given plate to get an average normalizing factor for that plate. In this manner, the average inter-assay CV was 12.01% for telomere plates and 9.16% for *36B4* plates. Normalization was done independently for each of the 7 different amplification efficiency approaches. |
| % of samples repeated and % of samples failing QC and excluding from further analyses | 9/364 = 2.5% of samples repeated.  1/364 = 0.27% of samples failed QC and excluded from analyses. |
| Acceptable range of PCR efficiency for single copy gene and telomere primers | N/A given the aim of the work included understanding the role of PCR efficiency on assay precision and external validity. |
| ICCs of samples/study groups to address variability | N/A. No random selection of samples was rerun for the purposes of determining an ICC. |
| T/S ratio transformed to a z-score before comparison across methods/studies | N/A. No comparison across studies was conducted. |
| How samples nested within families were accounted for | Samples collected from different tissues of the same participant, different time points for the same participant (n=9), and samples collected from different generations or siblings within the same family were always assessed on the same qPCR run excepting occasions where samples were rerun (n=9). |

**Supplementary Figure S1. Sample flow and subsets used for analyses. A**. 2152 replicate reactions comprising the full sample following reruns and removal of outlier replicates as described in Supplementary Table S1. **B.** Replicate reactions were distinguished by amplification target and analyzed separately due to concerns in reaction chemistry. **C.** Technical replicates (3x) were clustered by sample ID for analyses of coefficient of variation across replicate T-estimates and replicate S-estimates presented in Supplementary Tables S2-S5. When outlier replicates were identified, sample-level estimates were calculated with duplicate values instead of triplicate values, as was the case for 15 T estimates and 11 S estimates. **D.** Calculated T-estimates and S-estimates were used to calculate T/S values for a total of 363 samples with available data for T estimates and S estimates. Analyses included data for all samples except one buccal sample that failed quality control criteria on original assay and rerun attempt. T/S ratios generated using different efficiency approaches were used in analyses presented in the main text and Supplementary Tables S6-S8.

| **Supplementary Table S2: CV Across Replicate Natural Log Transformed T Estimates Calculated Using Different Amplification Efficiencies** | | | | | | | |
| --- | --- | --- | --- | --- | --- | --- | --- |
|  | T_RotorGeneCurve_ | T_RotorGeneBatch_ | T_LinRegRep_ | T_LinRegSamp_ | T_LinRegPlate_ | T_LinRegBatch_ | T_LinRegCurve_ |
| **All Samples** | 0.43% (0.28) | 0.43% (0.28) | 4.09% (2.57) | 0.40% (0.23) | 0.40% (0.23) | 0.40% (0.23) | 0.40% (0.23) |
| **Leukocyte Only** | 0.46% (0.26) | 0.46% (0.26) | 3.82% (2.23) | 0.41% (0.23) | 0.41% (0.23) | 0.41% (0.24) | 0.41% (0.24) |
| **Buccal Only** | 0.41% (0.29) | 0.42% (0.30) | 4.16% (2.65) | 0.40% (0.23) | 0.40% (0.23) | 0.40% (23) | 0.40% (0.23) |
| *Values reported are Mean (Standard Deviation). | | | | | | | |

| **Supplementary Table S3: Test Statistics and p-values from Pairwise Comparisons of CV Across Replicate Natural Log Transformed T Estimates Calculated Using Different Amplification Efficiencies** | | | |
| --- | --- | --- | --- |
| **Contrast** | **All Samples** | **Leukocyte Only** | **Buccal Only** |
| T_RotorGeneCurve_ vs T_RotorGeneBatch_ | t_362_=-2.548, p=0.011 | t_93_=-1.491, p=0.139 | t_268_=-2.064, p=0.040 |
| T_RotorGeneCurve_ vs T_LinRegRep_ | t_362_=-26.717, p=1.24E-87 | t_93_=-14.46, p=1.59E-25 | t_268_=-22.696, p=2.37E-64 |
| T_RotorGeneCurve_ vs T_LinRegSamp_ | t_362_=1.632, p=0.104 | t_93_=2.1, p=0.038 | t_268_=0.734, p=0.464 |
| T_RotorGeneCurve_ vs T_LinRegPlate_ | t_362_=1.606, p=0.109 | t_93_=2.088, p=0.040 | t_268_=0.713, p=0.476 |
| T_RotorGeneCurve_ vs T_LinRegBatch_ | t_362_=1.617, p=0.107 | t_93_=1.974, p=0.051 | t_268_=0.768, p=0.443 |
| T_RotorGeneCurve_ vs T_LinRegCurve_ | t_362_=1.786, p=0.075 | t_93_=2.052, p=0.043 | t_268_=0.919, p=0.359 |
| T_RotorGeneBatch_ vs T_LinRegRep_ | t_362_=-26.706, p=1.37E-87 | t_93_=-14.452, p=1.65E-25 | t_268_=-22.689, p=2.51E-64 |
| T_RotorGeneBatch_ vs T_LinRegSamp_ | t_362_=1.751, p=0.081 | t_93_=2.166, p=0.033 | t_268_=0.828, p=0.409 |
| T_RotorGeneBatch_ vs T_LinRegPlate_ | t_362_=1.727, p=0.085 | t_93_=2.155, p=0.034 | t_268_=0.807, p=0.420 |
| T_RotorGeneBatch_ vs T_LinRegBatch_ | t_362_=1.74, p=0.083 | t_93_=2.044, p=0.044 | t_268_=0.865, p=0.388 |
| T_RotorGeneBatch_ vs T_LinRegCurve_ | t_362_=1.906, p=0.057 | t_93_=2.116, p=0.037 | t_268_=1.015, p=0.311 |
| T_LinRegRep_ vs T_LinRegSamp_ | t_362_=27.647, p=3.01E-91 | t_93_=14.818, p=3.239E-26 | t_268_=23.519, p=4.07E-67 |
| T_LinRegRep_ vs T_LinRegPlate_ | t_362_=27.647, p=3.02E-91 | t_93_=14.822, p=3.1835E-26 | t_268_=23.518, p=4.12E-67 |
| T_LinRegRep_ vs T_LinRegBatch_ | t_362_=27.58, p=5.46E-91 | t_93_=14.784, p=3.7634E-26 | t_268_=23.464, p=6.24E-67 |
| T_LinRegRep_ vs T_LinRegCurve_ | t_362_=27.585, p=5.24E-91 | t_93_=14.784, p=3.7692E-26 | t_268_=23.469, p=6.00E-67 |
| T_LinRegSamp_ vs T_LinRegPlate_ | t_362_=-1.057, p=0.291 | t_93_=-0.805, p=0.423 | t_268_=-0.764, p=0.445 |
| T_LinRegSamp_ vs T_LinRegBatch_ | t_362_=-0.665, p=0.507 | t_93_=-1.47, p=0.145 | t_268_=0.125, p=0.900 |
| T_LinRegSamp_ vs T_LinRegCurve_ | t_362_=0.992, p=0.322 | t_93_=-0.636, p=0.527 | t_268_=1.511, p=0.132 |
| T_LinRegPlate_ vs T_LinRegBatch_ | t_362_=-0.319, p=0.75 | t_93_=-1.334, p=0.185 | t_268_=0.487, p=0.627 |
| T_LinRegPlate_ vs T_LinRegCurve_ | t_362_=1.372, p=0.171 | t_93_=-0.41, p=0.683 | t_268_=1.842, p=0.067 |
| T_LinRegBatch_ vs T_LinRegCurve_ | t_362_=2.782, p=0.006 | t_93_=1.072, p=0.287 | t_268_=2.61, p=0.010 |
| *Test statistics reported from paired samples t-test. Differences NOT significant at Bonferroni adjusted ∝=0.05/21 = 0.00238 are indicated in grey text. | | | |

| **Supplementary Table S4: CV Across Replicate Natural Log Transformed S Estimates Calculated Using Different Amplification Efficiencies** | | | | | | | |
| --- | --- | --- | --- | --- | --- | --- | --- |
|  | S_RotorGeneCurve_ | S_RotorGeneBatch_ | S_LinRegRep_ | S_LinRegSamp_ | S_LinRegPlate_ | S_LinRegBatch_ | S_LinRegCurve_ |
| **All Samples** | 0.42% (0.29) | 0.42% (0.29) | 3.94% (2.17) | 0.41% (0.26) | 0.41% (0.26) | 0.41% (0.26) | 0.41% (0.26) |
| **Leukocyte Only** | 0.44% (0.29) | 0.44% (0.29) | 3.81% (2.17) | 0.46% (0.29) | 0.46% (0.29) | 0.46% (0.30) | 0.46% (0.30) |
| **Buccal Only** | 0.41% (0.29) | 0.41% (0.29) | 3.99% (2.16) | 0.39% (0.24) | 0.39% (0.24) | 0.39% (0.25) | 0.39% (0.24) |
| *Values reported are Mean (Standard Deviation). | | | | | | | |

| **Supplementary Table S5: Test Statistics and p-values from Pairwise Comparisons of CV Across Replicate Natural Log Transformed S Estimates Calculated Using Different Amplification Efficiencies** | | | |
| --- | --- | --- | --- |
| **Contrast** | **All Samples** | **Leukocyte Only** | **Buccal Only** |
| S_RotorGeneCurve_ vs S_RotorGeneBatch_ | t_362_=-3.608, p=3.52E-04 | t_93_=-2.332, p=0.022 | t_268_=-2.797, p=0.006 |
| S_RotorGeneCurve_ vs S_LinRegRep_ | t_362_=-30.541, p=3.2667E-102 | t_93_=-14.834, p=3.02E-26 | t_268_=-26.693, p=1.87E-77 |
| S_RotorGeneCurve_ vs S_LinRegSamp_ | t_362_=0.83, p=0.407 | t_93_=-0.655, p=0.514 | t_268_=1.27, p=0.205 |
| S_RotorGeneCurve_ vs S_LinRegPlate_ | t_362_=0.749, p=0.454 | t_93_=-0.684, p=0.496 | t_268_=1.196, p=0.233 |
| S_RotorGeneCurve_ vs S_LinRegBatch_ | t_362_=0.686, p=0.493 | t_93_=-0.746, p=0.457 | t_268_=1.161, p=0.246 |
| S_RotorGeneCurve_ vs S_LinRegCurve_ | t_362_=0.885, p=0.376 | t_93_=-0.607, p=0.546 | t_268_=1.314, p=0.190 |
| S_RotorGeneBatch_ vs S_LinRegRep_ | t_362_=-30.533, p=3.5019E-102 | t_93_=-14.828, p=3.10E-26 | t_268_=-26.687, p=1.95E-77 |
| S_RotorGeneBatch_ vs S_LinRegSamp_ | t_362_=0.992, p=0.322 | t_93_=-0.537, p=0.592 | t_268_=1.393, p=0.165 |
| S_RotorGeneBatch_ vs S_LinRegPlate_ | t_362_=0.91, p=0.363 | t_93_=-0.567, p=0.572 | t_268_=1.318, p=0.189 |
| S_RotorGeneBatch_ vs S_LinRegBatch_ | t_362_=0.85, p=0.396 | t_93_=-0.628, p=0.532 | t_268_=1.286, p=0.199 |
| S_RotorGeneBatch_ vs S_LinRegCurve_ | t_362_=1.049, p=0.295 | t_93_=-0.486, p=0.628 | t_268_=1.438, p=0.152 |
| S_LinRegRep_ vs S_LinRegSamp_ | t_362_=31.774, p=9.4841E-107 | t_93_=15.222, p=5.46E-27 | t_268_=27.916, p=2.71E-81 |
| S_LinRegRep_ vs S_LinRegPlate_ | t_362_=31.761, p=1.0574E-106 | t_93_=15.216, p=5.61E-27 | t_268_=27.904, p=2.95E-81 |
| S_LinRegRep_ vs S_LinRegBatch_ | t_362_=31.674, p=2.199E-106 | t_93_=15.158, p=7.24E-27 | t_268_=27.838, p=4.72E-81 |
| S_LinRegRep_ vs S_LinRegCurve_ | t_362_=31.672, p=2.2345E-106 | t_93_=15.157, p=7.27E-27 | t_268_=27.836, p=4.79E-81 |
| S_LinRegSamp_ vs S_LinRegPlate_ | t_362_=-3.999, p=7.70E-05 | t_93_=-1.406, p=0.163 | t_268_=-3.835, p=1.57E-04 |
| S_LinRegSamp_ vs S_LinRegBatch_ | t_362_=-1.96, p=0.051 | t_93_=-0.864, p=0.390 | t_268_=-1.787, p=0.075 |
| S_LinRegSamp_ vs S_LinRegCurve_ | t_362_=0.406, p=0.685 | t_93_=0.457, p=0.648 | t_268_=0.174, p=0.862 |
| S_LinRegPlate_ vs S_LinRegBatch_ | t_362_=-1.033, p=0.302 | t_93_=-0.601, p=0.549 | t_268_=-0.838, p=0.403 |
| S_LinRegPlate_ vs S_LinRegCurve_ | t_362_=1.327, p=0.185 | t_93_=0.801, p=0.425 | t_268_=1.058, p=0.291 |
| S_LinRegBatch_ vs S_LinRegCurve_ | t_362_=3.647, p=3.05E-04 | t_93_=2.323, p=0.022 | t_268_=2.848, p=0.005 |
| *Test statistics reported from paired samples t-test. Differences NOT significant at Bonferroni adjusted ∝=0.05/21 = 0.00238 are indicated in grey text. | | | |

| **Supplementary Table S6: Test Statistics and p-values from Pairwise Comparisons of T/S Ratio Values Calculated Using Different Amplification Efficiencies** | | | |
| --- | --- | --- | --- |
| **Contrast** | **All Samples** | **Leukocyte Only** | **Buccal Only** |
| T/S_RotorGeneCurve_ vs T/S_RotorGeneBatch_ | t_362_=20.712, p=2.05E-63 | t_93_=10.368, p=3.42E-17 | t_268_=17.984, p=5.65E-48 |
| T/S_RotorGeneCurve_ vs T/S_LinRegRep_ | t_362_=32.122, p=5.14E-108 | t_93_=16.292, p=5.42E-29 | t_268_=27.637, p=1.99E-80 |
| T/S_RotorGeneCurve_ vs T/S_LinRegSamp_ | t_362_=33.785, p=5.71E-114 | t_93_=17.671, p=1.77E-31 | t_268_=28.759, p=6.78E-84 |
| T/S_RotorGeneCurve_ vs T/S_LinRegPlate_ | t_362_=40.141, p=2.30E-135 | t_93_=24.439, p=2.94E-42 | t_268_=32.695, p=1.61E-95 |
| T/S_RotorGeneCurve_ vs T/S_LinRegBatch_ | t_362_=40.125, p=2.59E-135 | t_93_=24.301, p=4.64E-42 | t_268_=32.725, p=1.32E-95 |
| T/S_RotorGeneCurve_ vs T/S_LinRegCurve_ | t_362_=-39.615, p=1.13E-133 | t_93_=-21.733, p=3.22E-38 | t_268_=-33.275, p=3.64E-97 |
| T/S_RotorGeneBatch_ vs T/S_LinRegRep_ | t_362_=31.846, p=5.19E-107 | t_93_=16.049, p=1.53E-28 | t_268_=27.468, p=6.74-80 |
| T/S_RotorGeneBatch_ vs T/S_LinRegSamp_ | t_362_=33.547, p=3.99E-113 | t_93_=17.451, p=4.33E-31 | t_268_=28.612, p=1.92E-83 |
| T/S_RotorGeneBatch_ vs T/S_LinRegPlate_ | t_362_=40.248, p=1.04E-135 | t_93_=24.444, p=2.88E-42 | t_268_=32.802, p=7.98E-96 |
| T/S_RotorGeneBatch_ vs T/S_LinRegBatch_ | t_362_=40.200, p=1.50E-135 | t_93_=24.269, p=5.15E-42 | t_268_=32.812, p=7.46E-96 |
| T/S_RotorGeneBatch_ vs T/S_LinRegCurve_ | t_362_=-40.445, p=2.46E-136 | t_93_=-22.306, p=4.21E-39 | t_268_=-33.899, p=6.49E-99 |
| T/S_LinRegRep_ vs T/S_LinRegSamp_ | t_362_=2.625, p=0.009 | t_93_=1.447, p=0.151 | t_268_=2.264, p=0.0244 |
| T/S_LinRegRep_ vs T/S_LinRegPlate_ | t_362_=4.799, p=2.00E-06 | t_93_=3.047, p=0.003 | t_268_=3.761, p=2.08E-04 |
| T/S_LinRegRep_ vs T/S_LinRegBatch_ | t_362_=4.482, p=1.00E-05 | t_93_=2.880, p=0.005 | t_268_=3.474, p=5.99E-04 |
| T/S_LinRegRep_ vs T/S_LinRegCurve_ | t_362_=-41.180, p=1.16E-138 | t_93_=-22.705, p=1.04E-39 | t_268_=-34.483, p=1.56E-100 |
| T/S_LinRegSamp_ vs T/S_LinRegPlate_ | t_362_=4.733, p=3.00E-06 | t_93_=3.174, p=2.04E-03 | t_268_=3.539, p=4.73EE-04 |
| T/S_LinRegSamp_ vs T/S_LinRegBatch_ | t_362_=4.368, p=1.60E-05 | t_93_=2.968, p=0.004 | t_268_=3.227, p=1.41E-03 |
| T/S_LinRegSamp_ vs T/S_LinRegCurve_ | t_362_=-41.922, p=5.53E-141 | t_93_=-23.464, p=7.67E-41 | t_268_=-34.948, p=8.27E-102 |
| T/S_LinRegPlate_ vs T/S_LinRegBatch_ | t_362_=-3.437, p=6.57E-05 | t_93_=-2.279, p=0.025 | t_268_=-2.614, p=0.009 |
| T/S_LinRegPlate_ vs T/S_LinRegCurve_ | t_362_=-43.732, p=1.55E-146 | t_93_=-25.520, p=8.80E-44 | t_268_=-36.049, p=8.68E-105 |
| T/S_LinRegBatch_ vs T/S_LinRegCurve_ | t_362_=-43.484, p=8.73E-146 | t_93_=-25.278, p=1.91E-43 | t_268_=-35.882, p=2.44E-104 |
| *Test statistics reported from paired samples t-test. Differences NOT significant at Bonferroni adjusted ∝=0.05/21 = 0.00238 are indicated in grey text. | | | |

| **Supplementary Table S7: Sample Coefficient of Variation for T/S Ratios Calculated Using Different Amplification Efficiencies** | | | | | | | |
| --- | --- | --- | --- | --- | --- | --- | --- |
|  | T/S_RotorGeneCurve_ | T/S_RotorGeneBatch_ | T/S_LinRegRep_ | T/S_LinRegSamp_ | T/S_LinRegPlate_ | T/S_LinRegBatch_ | T/S_LinRegCurve_ |
| **All Samples** | 46.20% | 46.08% | 111.11% | 100.00% | 42.47% | 40.54% | 43.46% |
| **Leukocyte Only** | 38.53% | 38.49% | 118.55% | 102.56% | 36.71% | 28.57% | 37.83% |
| **Buccal Only** | 48.86% | 48.73% | 102.22% | 93.10% | 45.07% | 43.06% | 45.47% |

| **Supplementary Table S8: Test Statistics and p-values from Pairwise Comparisons of Sample Coefficient of Variation for T/S Ratio Values Calculated Using Different Amplification Efficiencies** | | | |
| --- | --- | --- | --- |
| **Contrast** | **All Samples** | **Leukocyte Only** | **Buccal Only** |
| T/S_RotorGeneCurve_ vs T/S_RotorGeneBatch_ | $\chi_{1}^{2}$=1.03E-03, p=0.974 | $\chi_{1}^{2}$=2.25E-05, p=0.996 | $\chi_{1}^{2}$=1.36E=-03, p=0.971 |
| T/S_RotorGeneCurve_ vs T/S_LinRegRep_ | $\chi_{1}^{2}$=109.60, p=1.20E-25 | $\chi_{1}^{2}$=43.13, p=5.13E-11 | $\chi_{1}^{2}$=62.02, p=3.40E-15 |
| T/S_RotorGeneCurve_ vs T/S_LinRegSamp_ | $\chi_{1}^{2}$=93.33, p=4.42E-22 | $\chi_{1}^{2}$=38.42, p=5.69E-10 | $\chi_{1}^{2}$==52.56, p=4.18E-13 |
| T/S_RotorGeneCurve_ vs T/S_LinRegPlate_ | $\chi_{1}^{2}$=1.36, p=0.243 | $\chi_{1}^{2}$=0.235, p=0.628 | $\chi_{1}^{2}$=1.17, p=0.280 |
| T/S_RotorGeneCurve_ vs T/S_LinRegBatch_ | $\chi_{1}^{2}$=4.89, p=0.027 | $\chi_{1}^{2}$=2.32, p=0.128 | $\chi_{1}^{2}$=3.19, p=0.074 |
| T/S_RotorGeneCurve_ vs T/S_LinRegCurve_ | $\chi_{1}^{2}$=0.962, p=0.327 | $\chi_{1}^{2}$=0.027, p=0.868 | $\chi_{1}^{2}$=0.981, p=0.322 |
| T/S_RotorGeneBatch_ vs T/S_LinRegRep_ | $\chi_{1}^{2}$=110.12, p=9.21E-26 | $\chi_{1}^{2}$=43.09, p=5.2E-11 | $\chi_{1}^{2}$=62.50, p=2.66E-15 |
| T/S_RotorGeneBatch_ vs T/S_LinRegSamp_ | $\chi_{1}^{2}$=93.85, p=3.41E-22 | $\chi_{1}^{2}$=38.38, p=5.82E-10 | $\chi_{1}^{2}$=53.02, p=3.30E-13 |
| T/S_RotorGeneBatch_ vs T/S_LinRegPlate_ | $\chi_{1}^{2}$=1.29, p=0.256 | $\chi_{1}^{2}$=0.239, p=0.624 | $\chi_{1}^{2}$=1.09, p=0.296 |
| T/S_RotorGeneBatch_ vs T/S_LinRegBatch_ | $\chi_{1}^{2}$=4.75, p=0.029 | $\chi_{1}^{2}$=2.33, p=0.127 | $\chi_{1}^{2}$=3.06, p=0.080 |
| T/S_RotorGeneBatch_ vs T/S_LinRegCurve_ | $\chi_{1}^{2}$=0.90, p=0.343 | $\chi_{1}^{2}$=0.029, p=0.865 | $\chi_{1}^{2}$=0.910, p=0.340 |
| T/S_LinRegRep_ vs T/S_LinRegSamp_ | $\chi_{1}^{2}$=1.37, p=0.241 | $\chi_{1}^{2}$=0.537, p=0.464 | $\chi_{1}^{2}$=0.641, p=0.423 |
| T/S_LinRegRep_ vs T/S_LinRegPlate_ | $\chi_{1}^{2}$=128.92, p=7.07E-30 | $\chi_{1}^{2}$=47.43, p=5.70E-12 | $\chi_{1}^{2}$=76.69, p=2.00E-18 |
| T/S_LinRegRep_ vs T/S_LinRegBatch_ | $\chi_{1}^{2}$=146.93, p=8.12E-34 | $\chi_{1}^{2}$=56.85, p=4.69E-14 | $\chi_{1}^{2}$=86.83, p=1.19E-20 |
| T/S_LinRegRep_ vs T/S_LinRegCurve_ | $\chi_{1}^{2}$=125.78, p=3.44E-29 | $\chi_{1}^{2}$=44.59, p=2.43E-11 | $\chi_{1}^{2}$=75.43, p=3.79E-18 |
| T/S_LinRegSamp_ vs T/S_LinRegPlate_ | $\chi_{1}^{2}$=112.48, p=2.81E-26 | $\chi_{1}^{2}$=42.93, p=5.68E-11 | $\chi_{1}^{2}$=66.75, p=3.08E-16 |
| T/S_LinRegSamp_ vs T/S_LinRegBatch_ | $\chi_{1}^{2}$=130.58, p=3.06E-30 | $\chi_{1}^{2}$=52.95, p=3.42E-13 | $\chi_{1}^{2}$=76.68, p=2.01E-18 |
| T/S_LinRegSamp_ vs T/S_LinRegCurve_ | $\chi_{1}^{2}$=109.34, p=1.37E-25 | $\chi_{1}^{2}$=39.95, p=2.61E-10 | $\chi_{1}^{2}$=65.52, p=5.75E-16 |
| T/S_LinRegPlate_ vs T/S_LinRegBatch_ | $\chi_{1}^{2}$=1.10, p=0.295 | $\chi_{1}^{2}$=1.08, p=0.298 | $\chi_{1}^{2}$=0.501, p=0.479 |
| T/S_LinRegPlate_ vs T/S_LinRegCurve_ | $\chi_{1}^{2}$=0.035, p=0.852 | $\chi_{1}^{2}$=0.102, p=0.750 | $\chi_{1}^{2}$=0.008, p=0.928 |
| T/S_LinRegBatch_ vs T/S_LinRegCurve_ | $\chi_{1}^{2}$=1.52, p=0.217 | $\chi_{1}^{2}$=1.84, p=0.175 | $\chi_{1}^{2}$=0.636, p=0.425 |
| *Test statistics reported from paired samples t-test. Differences NOT significant at Bonferroni adjusted ∝=0.05/21 = 0.00238 are indicated in grey text. | | | |

| **Supplementary Table S9: Correlations Among T/S Ratios Calculated Using Different Amplification Efficiencies** | | | | | | |
| --- | --- | --- | --- | --- | --- | --- |
|  | T/S_RotorGeneCurve_ | T/S_RotorGeneBatch_ | T/S_LinRegRep_ | T/S_LinRegSamp_ | T/S_LinRegPlate_ | T/S_LinRegBatch_ |
| T/S_RotorGeneBatch_ | 0.999*** |  |  |  |  |  |
| T/S_LinRegRep_ | 0.100 | 0.102 |  |  |  |  |
| T/S_LinRegSamp_ | 0.162** | 0.162** | 0.957*** |  |  |  |
| T/S_LinRegPlate_ | 0.903*** | 0.911*** | 0.321*** | 0.376*** |  |  |
| T/S_LinRegBatch_ | 0.957*** | 0.960*** | 0.263*** | 0.328*** | 0.973*** |  |
| T/S_LinRegCurve_ | 0.937*** | 0.942*** | 0.289*** | 0.351*** | 0.987*** | 0.980*** |
| Correlations computed controlling for tissue. * p<0.05; **p<0.01; ***p<0.001 | | | | | | |

| **Supplementary Table S10: Correlation Between Age and T/S Ratios For Leukocyte and Buccal Samples** | | | |
| --- | --- | --- | --- |
| *Leukocyte Only* |  |  |  |
|  | **r** | **83.4% CI** | **Sample Size Needed for Power = 0.80** |
| **T/S_RotorGeneCurve_** | -0.20 | [-0.34 , -0.06] | 184.8 |
| **T/S_RotorGeneBatch_** | -0.20 | [-0.34 , -0.06] | 187.8 |
| **T/S_LinRegRep_** | 0.07 | [-0.08 , 0.21] | 1845.2 |
| **T/S_LinRegSamp_** | 0.07 | [-0.08 , 0.21] | 1698.1 |
| **T/S_LinRegPlate_** | -0.16 | [-0.30 , -0.02] | 291.3 |
| **T/S_LinRegBatch_** | -0.20 | [-0.33 , -0.05] | 200.3 |
| **T/S_LinRegCurve_** | -0.17 | [-0.31 , -0.02] | 271.1 |
| *Buccal Only* |  |  |  |
|  | **r** | **83.4% CI** | **Sample Size Needed for Power = 0.80** |
| **T/S_RotorGeneCurve_** | -0.17 | [-0.25 , -0.09] | 268.2 |
| **T/S_RotorGeneBatch_** | -0.17 | [-0.25 , -0.08] | 275.9 |
| **T/S_LinRegRep_** | -0.03 | [-0.11 , 0.06] | 11319.3 |
| **T/S_LinRegSamp_** | -0.06 | [-0.15 , 0.02] | 2104.1 |
| **T/S_LinRegPlate_** | -0.14 | [-0.22 , -0.06] | 393.2 |
| **T/S_LinRegBatch_** | -0.15 | [-0.23 , -0.07] | 339.6 |
| **T/S_LinRegCurve_** | -0.15 | [-0.23 , -0.06] | 366.0 |
| *Correlations computed controlling for sex | | | |

**Supplementary Figure S2: Results of simulation analyses**. Plot displays the results of 1000 simulation analyses to test for differences in correlation between TL and tissue relative to differences in correlation between TL and age with increasing noise in TL measurement. In this analysis two TL variables were created, an initial TL variable correlated with age at r=0.15 and tissue at 0.70, and a second ‘noisy’ TL variable correlated with the original TL variable at r≈0.87. After this the correlations between the noisy TL variable and age and tissue were assessed and compared to values observed for original TL variable to determine whether the correlation with tissue decreased more than the correlation with age when excess noise was introduced in the TL variable. This is expressed by the formula r_Non-noisy TL-Tissue_ – r_Noisy TL-Tissue_) – (r_Non-noisy TL-Age_ - r_Noisy TL-Age_). If this expression is positive, then the magnitude of improvement was greater for the correlation between TL and tissue than for the correlation between TL and age. This result was observed in 930 out of 1000 simulations and the mean value of the difference was 0.079±0.04.

**STATA Code for Simulation Analyses (Version 14.1)**

*this program is to simulate adding noise to a variable

*and observing what happens to correlations with a high and low correlation

*helpful for external validation measures that are more or less informative

{

clear

program drop noise // this line causes problems on the first run and needs to be omitted

program define noise, rclass

version 14.1

drop_all

matrix input m=(1,0.15,1,0.7,0.0,1) // making matrix with set correlation structure

drawnorm TL Age Tissue, n(200) corr(m) cstrorage(lower) clear

gen TL2=TL+runiform(-1,1) // adding noise to TL

*corr* // to double check correlation structure entered correctly—increase n to gain more precision – silenced to not slow down simulation

corr TL Age

local TL_Age = r(rho)

di ‘TL_Age’

corr TL2 Age

local TL2_Age = r(rho)

di ‘TL2_Age’

local age_diff=’TL_Age’ –‘TL2_Age’

di ‘age_diff’

corr TL Tissue

local TL_Tissue = r(rho)

di ‘TL_Tissue’

corr TL2 Tissue

local TL2_Tissue = r(rho)

di ‘TL2_Tissue’

local tissue_diff = ‘TL_Tissue’ – ‘TL2_Tissue’

di ‘tissue_diff’

gen tissue_age_diff = ‘tissue_diff’ – ‘age_diff’

sum tissue_age_diff

return scalar mean = r(mean)

end

simulate mean = r(mean), reps(10): noise //Set for 10 for diagnostics and then pushed up to 1,000 for more authoritative simulation analysis

sum mean

}
